# Berberine Synergistically Enhances the Antibacterial Activity of Ampicillin against Ampicillin-Resistant *Staphylococcus epidermidis*

**DOI:** 10.1021/acsomega.6c00665

**Published:** 2026-08-10

**Authors:** Htun Htun Win, Supphachai Onnom, Nipatchaya Sareephandon, Supa Plangklang, Kittipot Sirichaiwetchakoon, Surachat Buddhisa, Nattaphol Prakobkaew, Benjawan Dunkhunthod, Peerapat Krittanan, Yothin Teethaisong

**Affiliations:** † Department of Medical Sciences, Faculty of Allied Health Sciences, 37688 Burapha University, Chon Buri 20131, Thailand; ‡ Division of Pharmacology and Biopharmaceutical Sciences, Faculty of Pharmaceutical Sciences, Burapha University, Chon Buri 20131, Thailand; § Department of Medical Technology, Faculty of Allied Health Sciences, Burapha University, Chon Buri 20131, Thailand; ∥ Thai Traditional Medicine Program, Faculty of Nursing and Allied Health Sciences, 187409 Phetchaburi Rajabhat University, Phetchaburi 76000, Thailand

## Abstract

Although *S. epidermidis* is a skin commensal bacterium, it is thought to be a causative pathogen of skin infections. Prolonged and extensive use of antibiotics for skin infections, including ampicillin (AMP), has contributed to the increasing emergence of antibiotic-resistant bacteria and the shaping of skin commensal *S. epidermidis* into opportunistic pathogens. This highlights the urgent necessity for alternative therapeutic strategies to enhance antimicrobial efficiency and minimize toxic effects on host cells. The present study investigated the potential of berberine (BBR), with specific attention to AMP resistance and BBR as an antibiotic adjunct to conventional antibiotics against *S. epidermidis*. Minimum inhibitory concentration (MIC) was used to determine the antibacterial susceptibility profiles. BBR reduced the MIC of AMP against AMP-resistant *S. epidermidis* from 16 to 64 μg/mL to 2–16 μg/mL, and the fractional inhibitory concentration index (FICI) values of 0.3–0.5 confirmed the synergistic interactions of BBR and AMP in combination. A time-kill analysis of the BBR+AMP combination suggested bacteriostatic activity and synergistic interaction. An antibacterial resistance induction study of BBR and AMP, either alone or in combination, demonstrated no substantial change in MIC in BBR alone and BBR+AMP over 15 consecutive passages. *S. epidermidis* isolates harboring resistant genes determined by the multiplex polymerase chain reaction (PCR) revealed multiple resistance genes (*mecA*, *blaZ*, *aacA*, and *tetK*). The effects of the BBR+AMP combination were visualized using scanning electron microscopy (SEM) and exhibited abnormal septation and cell surface damage. Propidium iodide (PI) and SYTO-9 demonstrated that the BBR+AMP combination disrupted membrane integrity. The cytotoxicity effect of the combination of BBR+AMP on human keratinocyte skin cells (HaCaT) was determined by the MTT assay, which demonstrated no significant toxicity, and a therapeutic index (TI) analysis suggested an acceptable safety margin. These findings support combinations of BBR and AMP as alternative therapeutics for the treatment of skin infections caused by AMP-resistant *S. epidermidis*.

## Graphical abstract

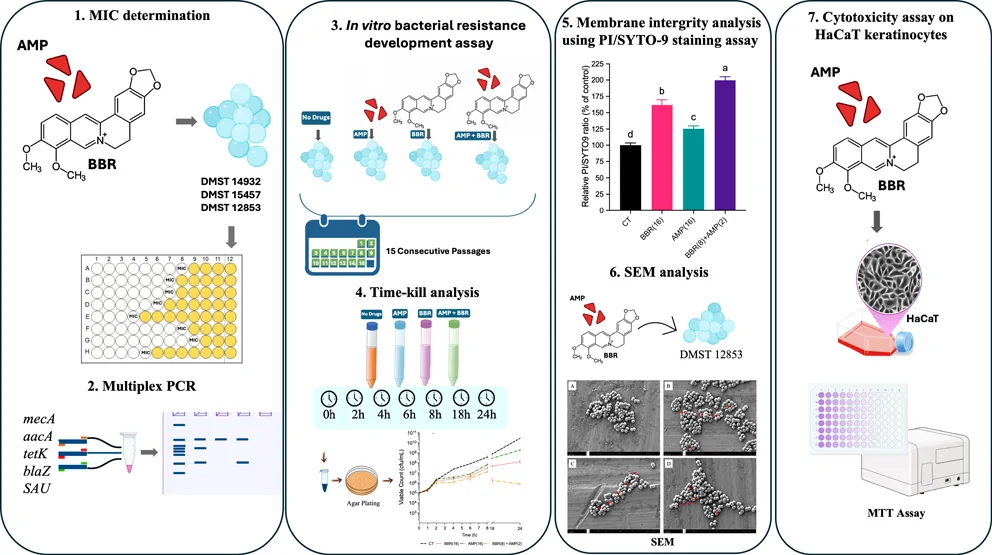

## 1 Introduction

*Staphylococcus epidermidis* (*S. epidermidis*) is considered a skin commensal bacterium. The metagenomic sequence data analysis revealed high functional diversity across the strains while maintaining overall community stability. Dysbiosis in microbiota diversity and functionality between skin microbiomes results in skin pathologic diseases. Conversely, a symbiosis of skin microorganisms plays a vital role in maintaining the skin barrier, modulating the immune system, and competitively suppressing the growth of pathogens.^1^ Due to dysbiosis conditions, *S. epidermidis* is capable of switching from commensal into an opportunistic bacterium, leading to skin infections and inflammation. Although certain *S. epidermidis* strains have several benefits such as inhibition of *S. aureus* biofilm formation and antiproliferative properties against cancer cells, the presence of antibiotic resistance genes may shift the skin commensal bacterium into a pathogen. In addition, *S. epidermidis* is known to exhibit multidrug and methicillin resistance, resulting in more complications in the selection of appropriate antibacterial chemotherapy.^2^ Infections caused by antibiotic-resistant *S. epidermidis* can be life-threatening.

Overconsumption and prolonged treatment of antibiotics for skin disorders have led to the development of antimicrobial resistance in skin commensal bacteria, including *S. epidermidis*. This issue has emerged as a major concern in dermatology.^3,4^ Moreover, increasing evidence of horizontal gene transfer among the closely related species of *Staphylococcus* spp. contributed to the transfer of antibiotic resistance genes across species.^5^ Staphylococcal Cassette Chromosome *mec* (SCC*mec*), specifically the *mecA* gene, is a common molecular mechanism of resistance in Staphylococci,^6^ while the *mecA* gene encodes a lower affinity of penicillin-binding proteins PBP2a for β-lactam antibiotics.^7^ Basically, ampicillin (AMP) has been clinically prescribed for the treatment of infections caused by the Gram-positive cocci, including *Staphylococcus* spp. However, prolonged administration of AMP has led to the increasing emergence of AMP resistance in *S. epidermidis* and *S. aureus.* Additionally, *blaZ*-encoded β-lactamase is also frequently identified in multidrug-resistant strains of *Staphylococcus* spp., particularly resistance to AMP and penicillin.^8^

Plant-derived antimicrobials are among the alternative strategies to conventional antibiotic therapy to overcome antibiotic resistance. In addition, using antibiotics alongside another drug or phytochemical can help to delay the development of bacterial resistance.^9^ Berberine (BBR) is an alkaloid compound and can be found in various medicinal plants, including goldenseal (*Hydrastis canadensis*), goldthread (*Coptis chinensis* or *Huanglian*), Oregon grape (*Berberis aquifolium*), tree turmeric (*Berberis aristata*), and European barberry (*Berberis vulgaris*).^10^ BBR possesses significant antimicrobial activity against a variety of bacteria^11^ and is found to have several pharmacological properties such as antiviral activities^10^ and anti-inflammatory properties.^13^ Remarkably, BBR is highly effective against Gram-positive bacteria such as *S. aureus* and *S. epidermidis* and presents moderate activity against Gram-negative bacteria.^14^ BBR showed strong synergistic antibacterial activity with linezolid, cefoxitin, and erythromycin, particularly against coagulase-negative Staphylococci (CoNS),^14^ whereas moderate synergistic antibacterial activities were exhibited against multidrug-resistant *Klebsiella pneumoniae* when combined with ciprofloxacin through the possible ciprofloxacin channel formation in the bacterial membrane.^15^ These findings highlight the potential of BBR and antibiotic synergistic properties with varying degrees to antibiotic classes and bacterial targets across the antibiotic mechanisms such as efflux pump function disruption, bacterial cell division disorder, and β-lactamase activity suppression.^16^

Despite BBR having received considerable attention, relatively few studies have focused on BBR, particularly in combination with antibiotic AMP to revive the antibacterial efficacy against AMP-resistant *S. epidermidis.* Similarly, other studies revealed the inhibitory properties of BBR when used with a combination of antibiotics such as penicillin, erythromycin, clindamycin, cefoxitin, ciprofloxacin, tobramycin, chloramphenicol, linezolid, tetracycline, and trimethoprim with sulfamethoxazole against certain CoNS.^14^ While the antimicrobial and resistance-modulating properties of individual BBRs have been reported, the evidence demonstrating the synergistic effect on bacterial growth and their cytotoxic effect on host cells remains undervalued. Therefore, this study aimed to address this gap by investigating the effectiveness of BBR in combination with AMP against AMP-resistant *S. epidermidis*.

## 2 Results and Discussion

### 2.1 MIC Determination and FICI of BBR with AMP Combination

The antibacterial susceptibility profiles of BBR and AMP against AMP-resistant *S. epidermidis* were investigated and indicated by the MIC values in Table 1. The MICs of BBR for *S. epidermidis* isolates are in the range from 32 to 64 μg/mL, while the MICs of AMP are 16–64 μg/mL. The highest resistance was seen in *S. epidermidis* DMST 14932. In drug combinations by a checkerboard assay, the concentration of BBR decreased from 32 and 64 μg/mL to 8 μg/mL against all three *S. epidermidis* isolates when BBR was used in combination. However, the MICs of AMP in combination were 16, 2, and 4 μg/mL toward *S. epidermidis* DMST 14932, 15457, and 12853, respectively. The FICI values of BBR and AMP in combination against *S. epidermidis* strains were found at 0.38, 0.31, and 0.50 against *S. epidermidis* DMST 14932, 15457, and 12853, respectively. These results indicated that BBR enhanced the antibacterial activity of AMP against AMP-resistant *S. epidermidis*, as shown in Table 2.

**Table 1:** Minimum Inhibitory Concentration of Berberine (BBR) and Ampicillin (AMP)

	MIC (μg/mL)	
bacterial isolates	BBR	AMP
*S. epidermidis* DMST 14932	64	64
*S. epidermidis* DMST 15457	32	32
*S. epidermidis* DMST 12853	32	16

**Table 2:** Drug Interaction of BBR in Combination with AMP against *S. epidermidis* Strains^a^

			checkerboard			
bacterial isolates	MIC_BBR_ in combination	MIC_AMP_ in combination	FIC_BBR_	FIC_AMP_	FICI	type of interaction
*S. epidermidis* DMST 14932	8	16	0.125	0.25	0.38	synergistic
*S. epidermidis* DMST 15457	8	2	0.25	0.0625	0.31	synergistic
*S. epidermidis* DMST 12853	8	4	0.25	0.25	0.50	synergistic

a Fractional inhibitory concentration index (FICI) ≤ 0.5 represents synergistic properties. FICI > 1.0 represents partial synergistic properties. FICI > 1.0 to ≤ 4.0 indicates indifference. FICI > 4.0 denotes antagonism.

While *S. epidermidis* infections are not typically lethal, increasing use and prolonged antibiotic treatment have resulted in the increasing emergence of antibiotic-resistant strains throughout the species, posing a significant public health concern. The isoquinoline alkaloid and amphipathic cation form of BBR is a potent substrate for multidrug resistance pumps (MDRs), which are membrane transporter proteins and can expel the toxins from the cells.^17^ In addition, BBR has been identified and concluded as a phospholipid bilayer penetrator, and the accumulation of BBR inside *S. aureus* is affected by blocking the MDR pump to prevent the extrusion of antibiotics or BBR.^18^ Furthermore, other *in vitro* and *in silico* studies demonstrated BBR as a potent efflux pump inhibitor that inhibits the major facilitator superfamily (MFS) efflux pump MdfA in *Escherichia coli*.^19^ Several studies have reported BBR as a promising antibiotic adjuvant for its enhancement properties over conventional antibiotics against *S. epidermidis* and *S. aureus* strains, indicating their synergistic properties.^20,21^ This aligns with our FICI results, which indicated a synergistic interaction between BBR and AMP, resulting in enhanced antibacterial activity.

### 2.2 *In Vitro* Bacterial Resistance Development Assay

Synergistic interaction between two antimicrobial agents may exhibit greater efficacy than monotherapy in combating antibiotic-resistant bacteria.^22^ However, the long-term effect of antimicrobial combination therapy on antibiotic resistance development remains insufficiently understood. The present study, therefore, investigated subinhibitory long-term exposure of BBR and AMP, either alone or in combination, on the induction of resistance in *S. epidermidis* over 15 consecutive passages. The MIC values of AMP alone progressively increased and increased up to a 128 μg/mLincrease after passage 13 in *S. epidermidis* 12853 (Figure 1A), whereas *S. epidermidis* 15457 exhibited increased resistance after passage 8 (Figure 1B). These findings indicate that prolonged exposure to subinhibitory concentrations of AMP alone could induce resistance in *S. epidermidis*. In contrast, the MIC value of BBR remained stable throughout 15 passages for both strains, suggesting sustained growth inhibition activity. The synergistic combination maintained stable MIC values for BBR and exhibited slight variation in the MIC of AMP after passage 13. At the endpoint (passage 15), the MIC of AMP alone was 128 μg/mL, while that in combination with BBR was 8 μg/mL. This substantial difference between MIC values of AMP monotherapy and in combination indicated the resistant nature of *S. epidermidis* toward AMP while highlighting the potential antimicrobial contribution of BBR as a potent adjuvant in combination treatment.

**Figure 1:**
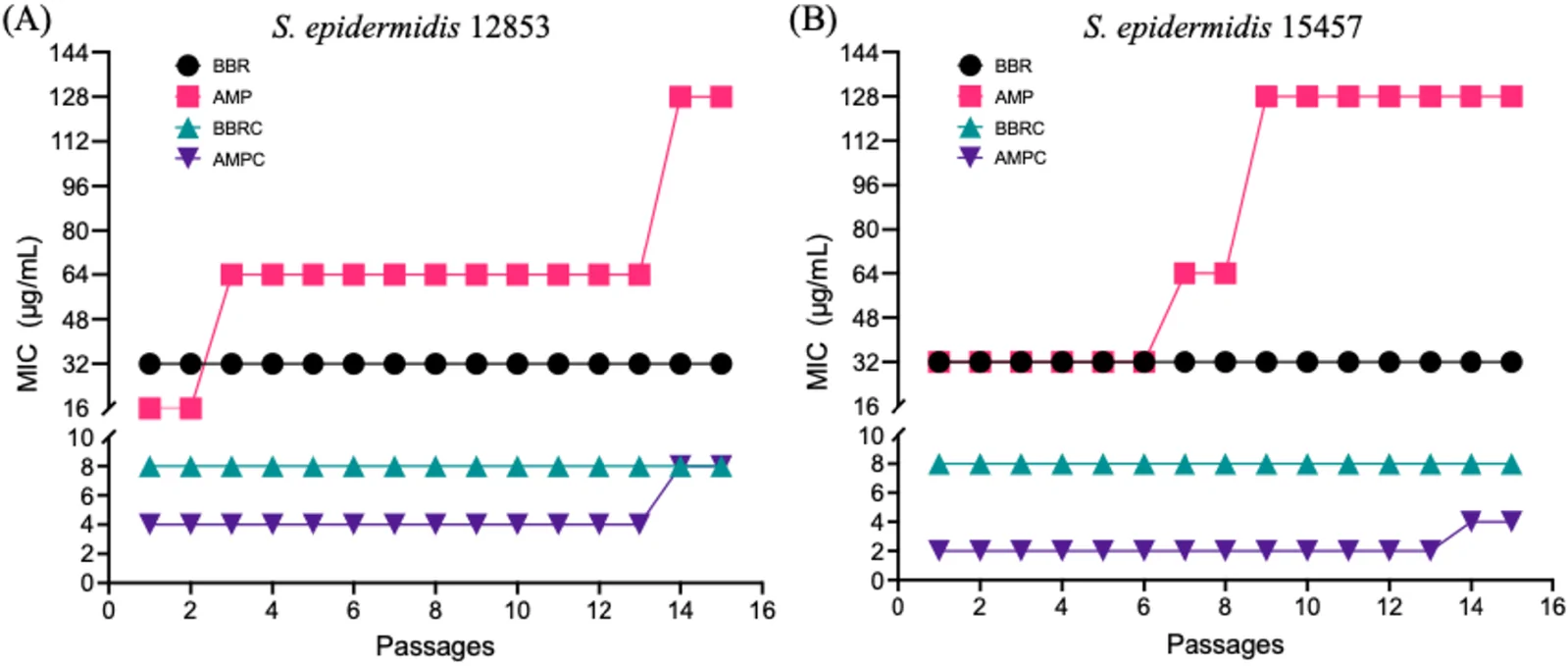
Changes in MIC values of BBR and AMP during 15 consecutive passages under subinhibitory combination treatment against *S. epidermidis* (BBRC: MIC value of BBR in combination; AMPC: MIC value of AMP in combination).

### 2.3 Time-Kill Kinetics Assay

Although the MIC and checkerboard assays are informative for antibacterial potency and interactions, both assays cannot provide the kinetic inhibition pattern and their antibacterial activity over time. Therefore, time-kill analysis was performed and bacterial viability of *S. epidermidis* 15457 monitored after exposure to subinhibitory concentrations of BBR and AMP individually, as well as to the synergistic combination of BBR 8/AMP 2 μg/mL. The untreated control exhibited continuous bacterial growth, reaching a population of 2.8 × 10^10^ cfu/mL after 24 h. In contrast, the BBR-treated group showed a reduction in bacterial growth to 1.4 × 10^8^ cfu/mL compared with the untreated control, whereas AMP-treated cells exhibited a slight reduction in the bacterial population of 1.9 × 10^9^ cfu/mL. The combination of BBR 8/AMP 2 μg/mL achieved a greater reduction than either BBR or AMP alone, decreasing the bacterial population to 8 × 10^5^ cfu/mL (Figure 2). This result indicates that the combination progressively reduced the number of viable bacteria over time.

**Figure 2:**
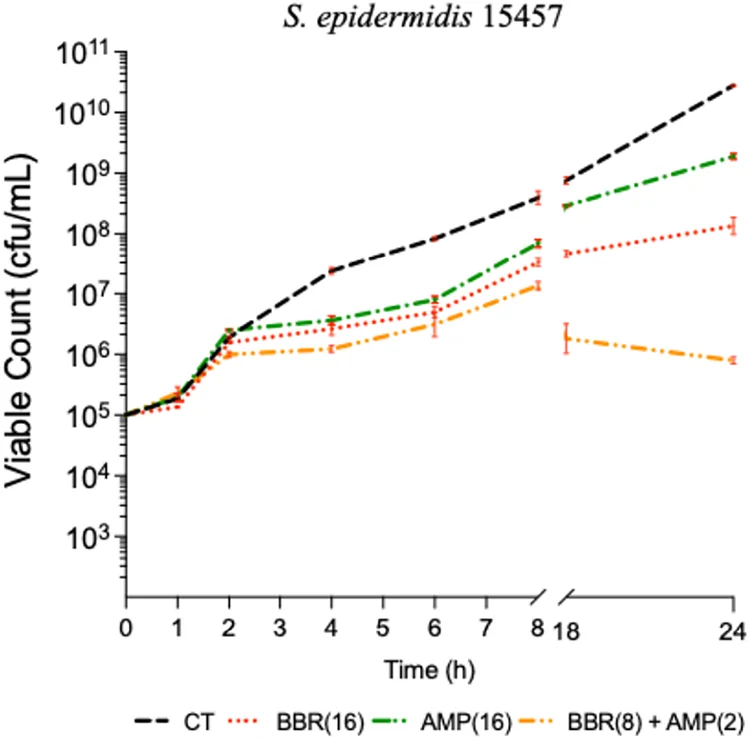
Time-kill kinetics analysis of the synergistic antibacterial activity of BBR and AMP against *S. epidermidis* 15457. Data are represented as mean ± SD of three independent replicates (*n* = 3).

The acceptable criterion for synergy in *in vitro* time-kill analysis is defined as a ≥2 log_10_ reduction in the bacterial population of combined treatment compared to monotherapy and is termed synergistic interaction, whereas a ≥3 log_10_ cfu/mL reduction is considered bactericidal.^23^ However, the time-kill study revealed a bacteriostatic effect toward *S. epidermidis* 15457 rather than bactericidal activity. In the present study, the bacteriostatic activity observed for BBR is consistent with previous studies that BBR bacteriostatically inhibited *S. epidermidis* growth.^24^ Notably, the combination treatment resurrected the antibacterial activity of AMP by suppressing bacterial growth in the time-kill analysis.

### 2.4 Multiplex PCR for Antibiotic Resistance Gene Detection

To detect antibiotic-resistant genes in *S. epidermidis* strains (DMST 12853, 15457, 14932) and one *S. aureus* strain ATCC 29213, a multiplex PCR method was employed to simultaneously observe the antibiotic resistance genes. Three isolates of *S. epidermidis* demonstrated the presence of *mecA* and *blaZ* genes, whereas they were absent from *S. aureus* 29213. The *aacA*-encoded aminoglycoside resistance was identified only in *S. epidermidis* DMST 14932. The *tetK*-encoded tetracycline resistance was seen in *S. epidermidis* 12853 and 15457. On the other hand, the *S. aureus*-specific gene *SAU*, an internal control, was exclusively observed in *S. aureus* 29213, while it was not present in *S. epidermidis* (Figure 3). Detection of multiple resistance-associated genes observed in *S. epidermidis* isolates indicated its multidrug-resistant characteristics. These findings further underscore the necessity for alternative therapeutic strategies, including antibiotic adjuvants such as BBR, against AMP-resistant *S. epidermidis* strains.

**Figure 3:**
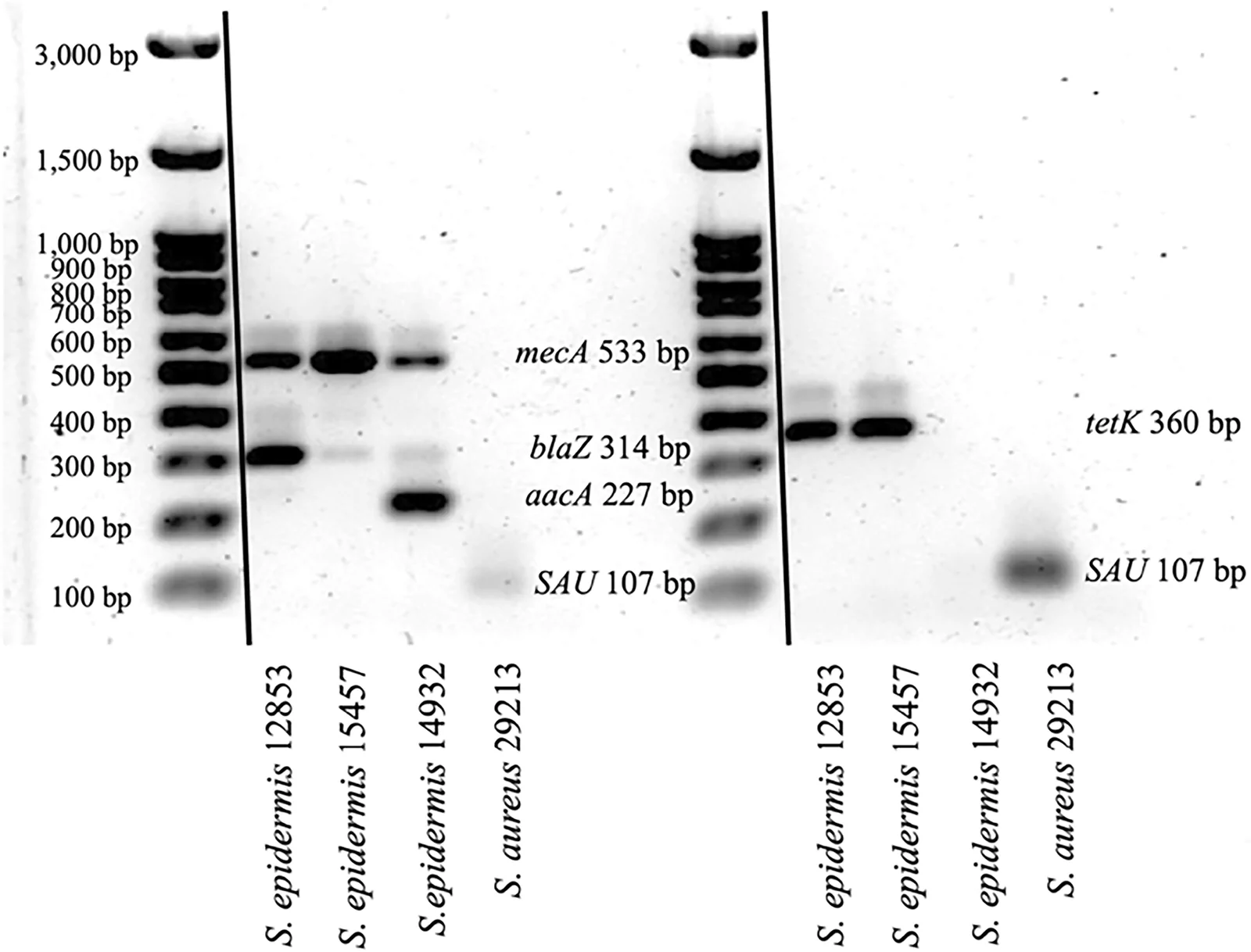
Agarose gel electrophoresis for the analysis of multiplex PCR for the detection of antibiotic resistance encoding genes (*mecA, blaZ, aacA, tetK, SAU*). The PCR amplicon separation was performed by 1.5% agarose gel electrophoresis. The 100 bp DNA ladder H3 RTU (Bio-Helix, Germany) was applied as a DNA marker.

### 2.5 Scanning Electron Microscope (SEM)

Cell morphological changes of *S. epidermidis* treated with BBR and AMP alone, and a combination of BBR and AMP were examined by SEM. Untreated control of *S. epidermidis* displayed intact spherical, uniform-sized, smooth cell walls and no damage on the surface of cell envelopes (Figure 4A). In contrast, the shape of the cells of the control treated with BBR resulted in a slightly rough cell surface, uneven size, and an increased number of diploid cells when compared to untreated bacteria, as shown in Figure 4B. Similarly, the alteration in bacterial cell walls, surfaces, sizes, and cell divisions were observed in the AMP-treated control (Figure 4C). Furthermore, the SEM results on the combined effects of BBR and AMP on *S. epidermidis* (Figure 4D) indicated similar morphological changes as BBR- or AMP-treated alone.

**Figure 4:**
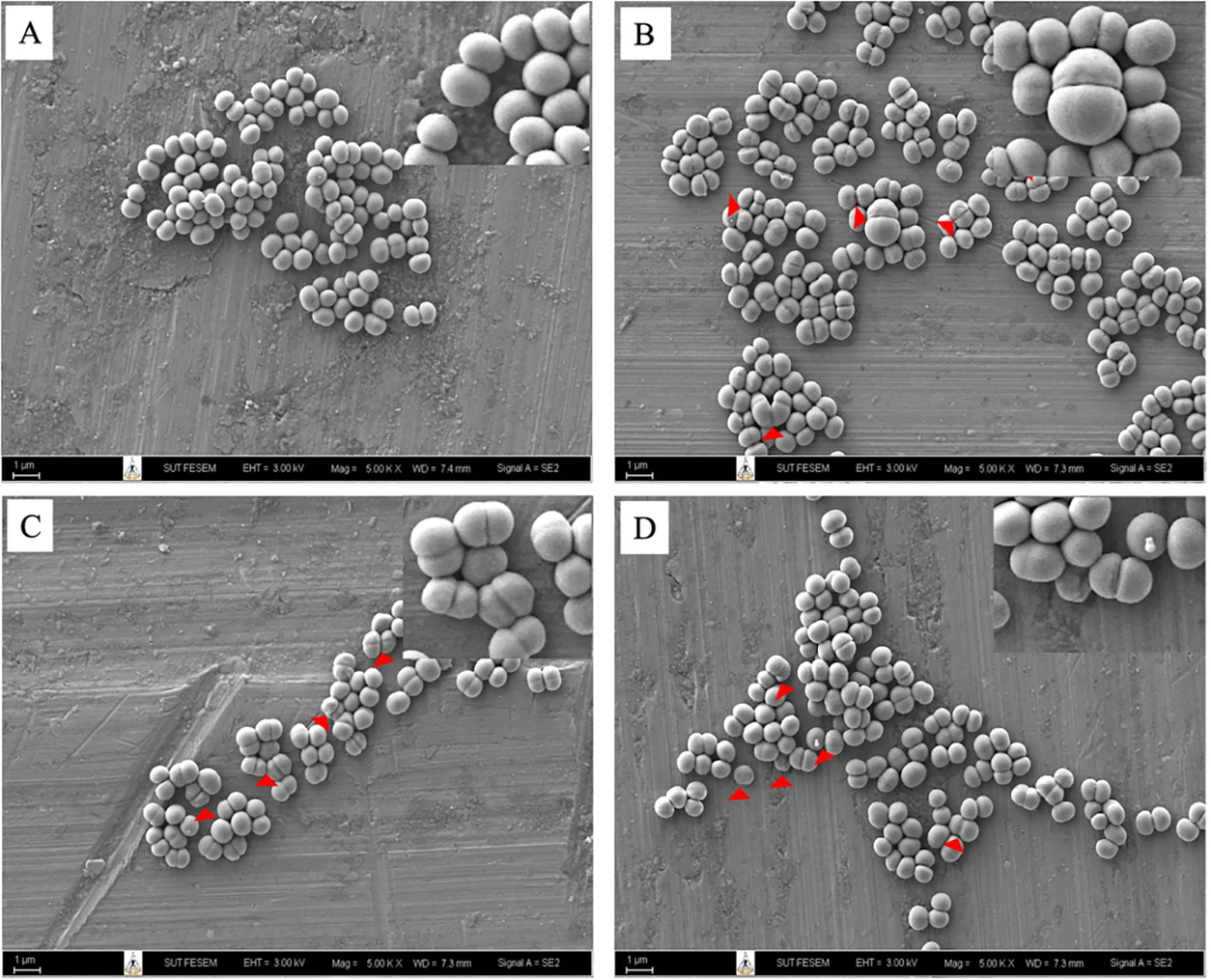
SEM of *S. epidermidis* DMST 12853 (A) untreated control, (B) treated control with BBR, (C) treated control with AMP, and (D) treated with the combination of BBR and AMP.

The SEM results showed that the morphology and ultrastructure of *S. epidermidis* in the control group showed normal morphological features, while the cell membranes and morphologies of the groups treated with BBR, AMP, and a combination of BBR and AMP showed cell damage. The irregular and incomplete septations were observed in treated groups, suggesting abnormal cell division. Antibiotic treatment of live bacteria (*Staphylococcus* species) hinders the cell division process via filamentous temperature-sensitive protein Z (FtsZ), which is essential for the cell division process.^13^ Furthermore, other studies revealed an asymmetrical cell morphology and the formation of abnormal septa during cell division in methicillin-resistant *S. aureus*.^26^

A previous study demonstrated that BBR accumulated within bacterial cells and downregulated the efflux pump, as well as disrupted bacterial membrane integrity, resulting in potassium leakage and dissipation of the proton motive force.^18^ Similarly, the collapse of the proton motive force has been reported to impair efflux pump function and potentially lead to drug accumulation.^19^ A recent study utilizing SEM and TEM (transmission electron microscopy) analyses demonstrated that BBR causes changes in MRSA cell morphologies, consistent with the disruption of membrane integrity after the treatment.^27^

### 2.6 Effects of BBR and AMP on Bacterial Membrane Integrity

The increasing PI/SYTO-9 fluorescence ratio reflects the proportion of compromised membranes because PI penetrates cells with deteriorated membranes, whereas SYTO-9 stains both intact and damaged cells. Therefore, the membrane integrity of *S. epidermidis* 15457 was assessed by using PI and SYTO-9 fluorescence staining. The PI/SYTO-9 fluorescence ratio was evaluated after normalization to the control group and was expressed relative to that of the control (CT) group. A higher normalized PI/SYTO-9 percentage indicates greater membrane damage and permeability, whereas a lower percentage indicates less damage and permeability. As shown in Figure 5, the relative PI/SYTO-9 ratio in the BBR-treated group increased to 160% of that of the control, while that in the AMP-treated group marginally increased to 120%. The highest relative fluorescence ratio was observed in the combination treatment group, which reached up to 200%. This indicates that membrane-damaging activity was potentially induced to a greater extent by the combination treatment than by either BBR or AMP alone. In addition, the elevated relative PI/SYTO-9 fluorescence ratio observed in the combination group may suggest an increased membrane permeability, thereby enhancing the PI uptake into bacterial cells. This finding corresponds to a time-kill curve analysis showing that the combination dramatically reduced bacterial viability compared with the monotreatment.

**Figure 5:**
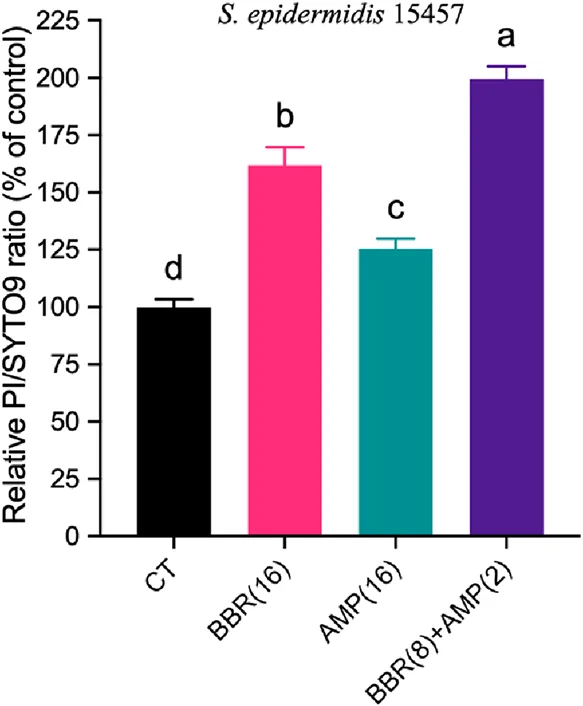
Effect of BBR, AMP, and combination treatment on the membrane integrity of *S. epidermidis* determined by normalized PI/SYTO-9 fluorescence ratio. The mean ± SD for three replicates is illustrated, and the relative viability is expressed as a percentage relative to the untreated control (CT). The same subscripts are not significantly different from each other (Tukey’s multiple comparison test, *p* < 0.05).

Likewise, this observation may also contribute to enhancing antibacterial activity and indicates membrane disruption as one of the mechanisms. Zhang et al. (2020) reported that BBR caused a fluctuation in lipid profile and altered the cell membrane integrity of *S. aureus*,^28^ while Zhao et al. (2023) suggested that membrane disruption was associated with the cation penetration of BBR into phospholipid bilayers.^18^

### 2.7 MTT Assay

The cytotoxic properties of BBR in combination with AMP were first evaluated on HaCaT keratinocytes. On the other hand, BBR at a higher dose has been shown to exert dose-dependent cytotoxic effects on human cell lines, which was reported in topical application and suppressive properties of BBR on skin inflammation.^29^ In addition, BBR concentrations higher than 50 μg/mL caused an obvious decline in the viability of murine fibroblast L929 cells and altered the cell morphology at 100 μg/mL.^30^ In contrast to BBR cytotoxicity, MTT assay results from HaCaT keratinocytes treated with AMP alone showed no significant cell reduction up to 32 μg/mL of AMP. As shown in Figure 6A, cell viability remained at 100% at the highest concentration of AMP. Therefore, nontoxic concentrations were selected as the safe and effective dosage range for subsequent experiments, and a combination of BBR and AMP was used for HaCaT cell viability.

**Figure 6:**
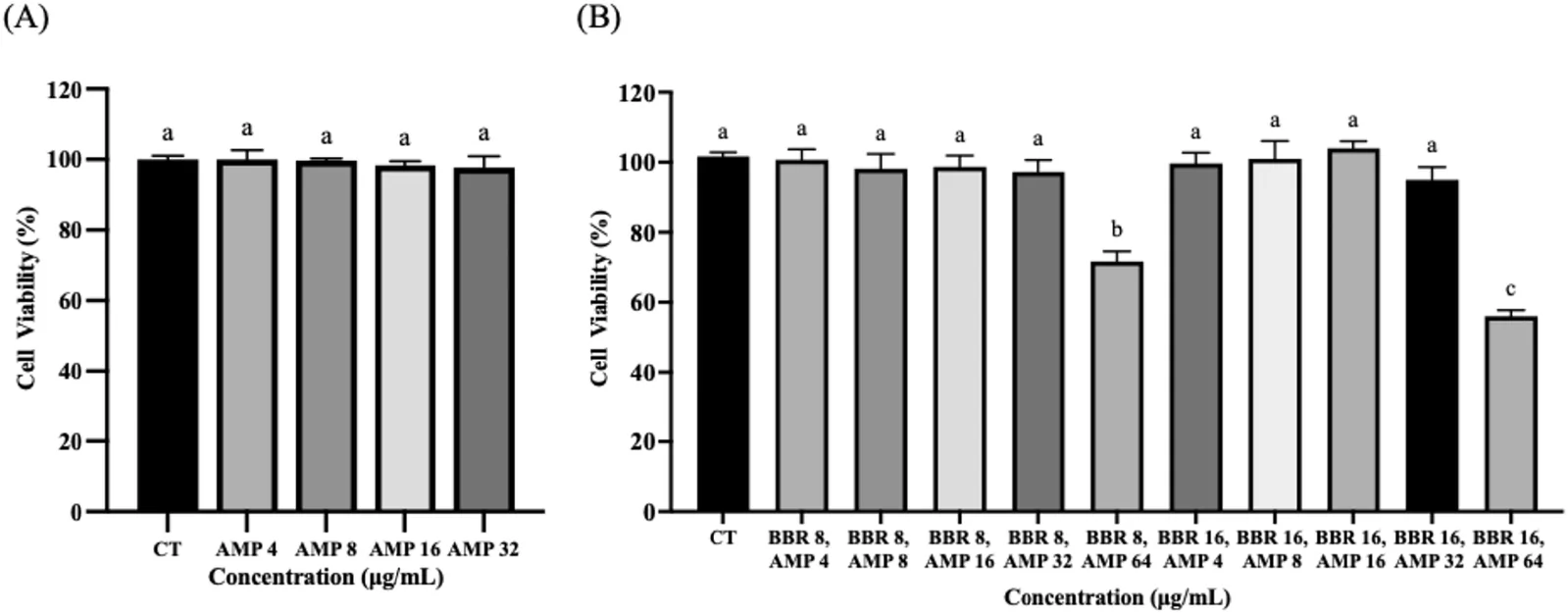
Cytotoxicity of (A) AMP and (B) BBR and AMP in combination with HaCaT keratinocytes. The mean ± SD for six replicates is illustrated, and cell viability is expressed as a percentage relative to untreated control cells (CTs). The same subscripts are not significantly different from each other (Tukey’s multiple comparison test, *p* < 0.05).

The cytotoxic properties of BBR combined with AMP showed a significant reduction in cell viability at higher concentration combinations of AMP, such as BBR 8/AMP 64 μg/mL and BBR 16/AMP 64 μg/mL (Figure 6B). Notably, cell viability at a lower concentration of a combination of BBR 8 μg/mL with AMP 4–32 μg/mL and BBR 16 μg/mL with AMP 4–32 μg/mL remained close to untreated control cells with no statistically significant differences. In contrast, increasing the AMP concentration in the presence of BBR further declined cell viability, indicating that the combination led to a concentration-dependent reduction in cell viability. Importantly, this suggests that treating the cells with a combination of BBR and high doses of AMP may contribute to increased cytotoxic effects on HaCaT cells.

To evaluate the therapeutic index (TI), the effect of the 50% cytotoxic concentration (CC_50_) value of the BBR + AMP combination on HaCaT cells was divided by the MIC value. A TI > 10 was considered to indicate pharmacological safety.^31^ The CC_50_ of varying concentrations of AMP in combination with BBR at 8 μg/mL was approximately 82.6 μg/mL, whereas the CC_50_ of combination with BBR at 16 μg/mL was 67.4 μg/mL. The combinations exhibiting a TI above 10 were BBR 8/AMP 4 μg/mL, BBR 8/AMP 8 μg/mL, and BBR 16/AMP 4 μg/mL, with TI values of 20.7, 10.3, and 16.9, respectively. Collectively, the combination treatment reduced AMP concentrations while maintaining acceptable TI values above 10. The combination with relatively close concentrations of the two antimicrobial agents may possess greater potential to minimize the risk of resistance development during prolonged treatment.^32^ This finding supports the promising application of a lower dose of the combination of BBR and AMP for topical application while maintaining antibacterial efficacy.

## 3 Conclusions

The FICI values obtained from a combination of BBR and AMP demonstrated a synergistic interaction against AMP-resistant *S. epidermidis*. Notably, BBR enhanced the antibacterial efficacy of AMP and maintained stable antimicrobial activity over 15 passages at subinhibitory concentrations of the monotherapy and combination treatment. Kinetic growth inhibition by time-kill analysis demonstrated that the BBR+AMP combination dramatically inhibited *S. epidermidis* viability. SEM analysis provides evidence that treatment with BBR and AMP, either alone or in combination, induced cell disruption and structural deformation in *S. epidermidis*. Membrane integrity determined by PI and SYTO-9 staining indicated that the BBR+AMP combination disrupted the membrane integrity of *S. epidermidis*. The MTT assay results demonstrated the concentration-dependent cytotoxicity of the combination treatment, whereas AMP alone exhibited no cytotoxic effects toward HaCaT keratinocytes. Importantly, TI values indicated acceptable pharmacological safety in BBR and AMP combinations. Taken together, the findings from the present study suggest that BBR acts as a potent antibiotic adjuvant capable of enhancing AMP susceptibility and potentially minimizing resistance development during prolonged treatment. Therefore, the BBR and AMP combination may represent a novel, promising therapeutic candidate for the treatment of infections caused by AMP-resistant *S. epidermidis*. Further investigations, including the concurrent interaction of BBR and AMP with the underlying molecular mechanisms of membrane disruption and toxicity in animal and human models, are warranted.

## 4 Methods

### 4.1 Bacterial Strains and Antibiotics

The bacterial strains employed in the present study included *S. epidermidis* DMST 12853, *S. epidermidis* DMST 14932, and *S. epidermidis* DMST 15457. These were obtained from the Department of Medical Science of Thailand (DMST), Thailand. A reference strain of *S. aureus* ATCC 29213 was obtained from the American Type Culture Collection. BBR and AMP antibiotics were acquired from Sigma-Aldrich, Singapore. BBR was prepared for dissolution in deionized water and filtered through a 0.22 μm CA syringe filter (Pall Corporation, UK).

### 4.2 Minimum Inhibitory Concentration (MIC)

The MIC for antimicrobial susceptibility profile was determined by the broth microdilution assay in accordance with CLSI (Clinical and Laboratory Standards Institute) standard guidelines.^33^ To determine MIC values, BBR and antibiotic AMP were prepared according to the manufacturer’s instructions. An overnight bacterial culture was collected, washed, and adjusted to 0.5 McFarland equivalent, followed by diluting the suspension to obtain 5 × 10^6^ cfu/mL prior to adding 20 μL of diluted bacteria to 180 μL of cation-adjusted Mueller–Hinton broth (CAMHB) medium with serial concentrations of BBR or AMP. Control wells were free from bacteria and antibiotics. The MIC results after incubation for 18 h at 37 °C were investigated by observing turbidity and examining the microplate reader at a wavelength of 600 nm. The lowest concentration showing no growth was designated as the MIC.^34^ The experiment was performed in three independent replications.

### 4.3 Determination of Drug Interaction by Checkerboard Assay

The drug interaction was evaluated using a checkerboard assay, which indicated the fractional inhibitory concentration index (FICI). The experiments were performed by following previous studies with slight modifications.^35,36^ A combination of BBR and AMP was prepared to investigate the synergistic interaction against three *S. epidermidis* strains, and the experiment was carried out similarly to that performed for the MIC determination. In brief, 20 μL of bacterial suspension, 20 μL of serial concentrations of antibiotics, and 20 μL of different concentrations of BBR were added to 140 μL of the CAMHB medium to obtain a final volume of 200 μL/well. Subsequently, the microplates were incubated overnight at 37 °C. The FICI was evaluated by the following equation

$$\mathrm{FICI}={\mathrm{FIC}}_{A}+{\mathrm{FIC}}_{B}=\frac{\mathrm{Conc. of A in MICs of A + B}}{\mathrm{MIC of A alone}}+\frac{\mathrm{Conc. of B in MICs of A + B}}{\mathrm{MIC of B alone}}$$

where FICI ≤ 0.5 represents synergistic properties, FICI > 1.0 indicates partial synergistic properties, FICI > 1.0 to ≤ 4.0 denotes indifference, and FICI > 4.0 indicates antagonism.

### 4.4 *In Vitro* Bacterial Resistance Development Assay

The MIC antimicrobial susceptibility profile shows that single colonies of *S. epidermidis* 12853 and 15457 were inoculated into the CAMHB medium containing subinhibitory concentrations of the drugs either in combination or alone by following the previously described method with slight modifications.^31^ Overnight cultures of *S. epidermidis* 12853 and 15457 were adjusted to 0.5 McFarland standard. The bacterial suspension was further diluted to achieve 5 × 10^5^ cfu/mL in the presence of subinhibitory concentrations of the BBR and AMP monotherapy, as well as combination therapy. Following incubation at 37 °C for 18 h, bacterial cells were collected by centrifugation for MIC determination, as described in Section 4.2. The experiments continued for 15 consecutive passages. Bacterial resistance induction was monitored using MIC values obtained from each passage, and the data were presented as BBR, AMP, and combination treatment values represented by BBRC and AMPC.

### 4.5 Time-Kill Analysis Assay

The antibacterial and synergistic properties of BBR and AMP, either alone or in combination, against *S. epidermidis* 15457 were assessed using the time-kill assay following a previously reported method.^37,38^ Initially, the optical density of the bacterial inoculum was adjusted to 5 × 10^5^ cfu/mL. The bacteria were exposed to subinhibitory concentrations of BBR and AMP individually, the combination of BBR 8/AMP 2 μg/mL, or no antibacterial agents as the untreated control, and incubated for 0, 1, 2, 4, 6, 8, 18, and 24 h. Following incubation, the samples were collected, and 100 μL of each sample was serially diluted with 900 μL of normal saline in a microcentrifuge tube. Subsequently, 10 μL of each diluted suspension was directly dropped onto a cation-adjusted Mueller–Hinton agar (CAMHA) medium and incubated overnight at 37 °C. The resulting colonies (cfu/mL) were enumerated according to the following formula: mean colony count × dilution factor × 100. Synergistic activity was defined as a reduction of ≥2 log_10_ cfu/mL in bacterial count, whereas bactericidal activity was defined as a reduction of ≥3 log_10_ cfu/mL relative to the initial inoculum.^23^

### 4.6 Detection of Antibiotic-Resistant Genes by Multiplex PCR

The presence of antibiotic-resistant genes such as *mecA, aacA*, *tetK, blaZ*, and *SAU* was investigated using a multiplex PCR technique following previous studies with slight modifications. The primer sequences and amplicon sizes are listed in Table 3.^39^ Briefly, bacterial genomic DNA was isolated from bacterial cultures grown overnight using the GF-1 bacterial DNA extraction kit (Vivantis, Malaysia) according to the manufacturer’s instructions. Multiplex PCRs were carried out using GoTaq Green Master Mix (Promega Corporation, USA) comprising 100 ng of DNA template, green master mix, a mixture of primers, and nuclease-free water. PCR amplification was performed using a Bio-Rad T100 thermal cycler, and the thermocycling conditions were set as follows: initial denaturation step at 95 °C for 5 min, followed by 30 cycles of denaturation at 95 °C for 30 s, annealing at 60 °C for 30 s, and extension at 72 °C for 1 min, with a final extension at 72 °C for 5 min. The PCR products were analyzed by 1.5% agarose gel electrophoresis containing SYBR Safe DNA staining dye and run at 100 V for 45 min. The DNA bands were visualized and documented using a Chemidoc Touch Image system (Bio-Rad, USA).

**Table 3:** Primer Sequences for Antibiotic-Resistant Genes Detected in *S. epidermidis* and *S. aureus*

target genes	sequences (5′→3′)	amplicon size (bp)
*mecA_F*	AAA ATC GAT GGT AAA GGT TGG C	533
*mecA_R*	AGT TCT GCA GTA CCG GAT TTG C	
*aacA_F*	TAA TCC AAG AGC AAT AAG GGC	227
*aacA_R*	GCC ACA CTA TCA TAA CCA CTA	
*tetK_F*	GTA GCG ACA ATA GGT AAT AGT	360
*tetK_R*	GTA GTG ACA ATA AAC CTC CTA	
*blaZ_F*	AAC ACC TGC TGC TTT CGG TA	314
*blaZ_R*	CAC TCT TGG CGG TTT CAC TT	
*SAU_F*	AAT CTT TGT CGG TAC ACG ATA TTC TTC ACG	107
*SAU_R*	CGT AAT GAG ATT TCA GTA GAT AAT ACA ACA	

### 4.7 Scanning Electron Microscope (SEM)

The samples were prepared and processed by following minor modifications of the method described by a previous study.^37^ The morphological changes of *S. epidermidis* treated with BBR, AMP, and a combination of BBR and AMP were visualized by SEM. Log-phase bacterial cultures were treated with BBR, AMP, or a combination of BBR and AMP. Untreated cultures served as the control. The samples were collected by centrifugation at 6000 rpm for 15 min and fixed with 2.5% (w/v) glutaraldehyde overnight at 4 °C. Thereafter, the samples were washed three times with phosphate-buffered saline, and 1% osmium tetroxide was applied for staining for 2 h. The samples were centrifuged at 8000 rpm for 5 min after washing with distilled water three times. Cell dehydration processes were carried out using graded acetone (20%, 40%, 60%, 80%, and 100%). The glass slides were used to transfer the cells for the air-drying processes. The cell morphologies were captured with a scanning electron microscope (Zeiss Leo 1450VP SEM, England) after the samples were attached on stubs using carbon tape and coated with gold.

### 4.8 Membrane Integrity Analysis with the PI-SYTO-9 Staining Assay

Individual and combined effects of BBR and AMP on membrane integrity were evaluated using propidium iodide (PI) and SYTO-9 staining methods with slight modifications to the previously described method.^41^ *S. epidermidis* 15457 was grown to the log phase in the CAMHB medium containing subinhibitory concentrations of BBR, AMP, or a combination of antibacterial agents (BBR 8/AMP 2 μg/mL), while the untreated control was incubated without BBR and AMP. Subsequently, bacterial suspensions were collected and washed with phosphate-buffered saline (PBS, pH 7.4), and the bacterial pellets were resuspended in 1 mL of PBS. Then, the optical density of the suspension was adjusted to OD600 0.5, and the cell pellets were collected by centrifugation. Next, the bacterial cells were mixed with 200 μL of dye mixture containing PI and SYTO-9 at final concentrations of 1 μM and 5 μM, respectively, and incubated at 4 °C in the absence of light. The samples were then washed three times and resuspended in 200 μL of PBS. Subsequently, 50 μL of each resuspended sample was transferred into a black 96-well microplate, and fluorescence intensities were measured at excitation/emission wavelengths of 488/530 nm (green) and 538/617 nm (red) using a fluorescence microplate reader (EnSight, PerkinElmer, USA).

### 4.9 Cell Culture

A human skin cell line, HaCaT keratinocyte, was acquired from Cell Lines Service GmbH (CLS, Eppelheim, Germany). The HaCaT keratinocyte cells were cultured in Dulbecco’s modified Eagle’s medium (DMEM) (Gibco, Billings, MT) and incubated at 37 °C in a 5% CO_2_ incubator containing a humidified atmosphere. Complete DMEM was prepared with 10% fetal bovine serum (FBS), 1.5% HEPES, and 1% penicillin–streptomycin antibiotics.

### 4.10 Cytotoxicity and Therapeutic Index (TI) of BBR and AMP Combinations in HaCaT Cells Determined by the MTT Assay

HaCaT keratinocyte cells were seeded at a density of 1.5 × 10^4^ cells/well in a 96-well plate (Corning Life Sciences, Wujiang, China) and incubated at 37 °C in a 5% CO_2_ incubator for 24 h. For the treatment, BBR, AMP, and the combination were prepared and incubated for 24 h before subsequent assays. After 24 h of treatment with BBR, antibiotics alone, and in combination, the culture medium was removed, and 100 μL of the MTT solution (0.5 mg/mL) was added to each well. After the plate was further incubated for 4 h at 37 °C, the MTT solution was removed, and the formazan crystal formed was solubilized with 100 μL of 100% DMSO. The results were measured and read at 570 nm, and cell viability was calculated as a percentage compared to the untreated control cells. A total of six replications were carried out for cytotoxicity experiments.

The therapeutic index (TI) of the combined agents was calculated based on a previous study.^38,42^ The CC_50_ value was obtained from the fitted dose–response curve. TI was calculated using the following formula

$$\mathrm{TI}={\mathrm{CC}}_{50}\mathrm{of AMP in combination}/\mathrm{MIC of AMP in combination}$$

where CC_50_ and antimicrobial denominator (MIC) represent the specific concentrations of the drug AMP present within the fixed-ratio mixture of the combination.

### 4.11 Statistical Analysis

The one-way ANOVA statistical analysis was performed on the data from at least 3 independent experiments and expressed as mean ± SD. The statistically significant differences (*p* value < 0.05) were assessed using Tukey’s multiple comparison test on the GraphPad Prism program (Version 10, GraphPad Software, USA).
